# Shengmai Yin formula modulates the gut microbiota of spleen-deficiency rats

**DOI:** 10.1186/s13020-020-00394-y

**Published:** 2020-10-28

**Authors:** Yu You, Lin Luo, Yanyan You, Yanjun Lin, Huiling Hu, Yunhui Chen, Chaomei Fu, Tian Xie

**Affiliations:** 1 College of Pharmacy, Chengdu University of Traditional Chinese Medicine, Chengdu, Sichuan China; 2 Department of Pharmacy and Laboratory Medicine, Sichuan Nursing Vocational College, 173 Lung Du Nan Road, Chengdu, 610100 Sichuan China; 3 Holistic Integrative Pharmacy Institutes, Hangzhou Normal University, 2318# Yuhangtang Road, Cangqian Street, Yuhang District, Hangzhou, 31112 Zhejiang China

**Keywords:** Shengmai Yin formula, Spleen deficiency, Gut microbiota, 16S rRNA

## Abstract

**Background:**

Spleen-deficiency syndrome, an important pathological change in traditional Chinese medicine, has been proven to attribute to intestinal dysbacteriosis. Shengmai Yin (SMY), a classic formula for replenishing qi and restoring pulse, is a common medicine for critical emergencies in traditional Chinese Medicine. Interestingly, our previous study established a spleen-deficiency rat model and verified the potency of SMY formula in curing spleen-deficiency rats. Our goal herein was to explore whether SMY can modulate the composition of intestinal flora and alleviate spleen-deficiency in rats.

**Methods:**

This experiment was randomly divided into three groups, namely the normal control group (NC), model control group (MC), and the Shengmai Yin group (SMY). After the treatment, the weight and symptom indexes of the rats were recorded, histological changes in the colon were observed, levels of serum D-xylose, gastrin (GAS), and vasoactive intestinal peptide (VIP) were measured, and gut microbiota profiling was conducted by 16S rRNA sequencing.

**Results:**

The body mass of the spleen-deficiency model rats significantly decreased compared with that of the NC group, and SMY treatment significantly increased body mass compared with the MC group (*P* < 0.01). Colon histopathology revealed that SMY treatment alleviated colonic mucosal damage in spleen-deficiency rats. The serum levels of D-xylose and gastrin (GAS) were significant increased by SMY (*P* < 0.05, *P* < 0.01), and vasoactive intestinal peptide (VIP) was reduced by SMY (*P* < 0.01) compared with MC. Furthermore, alpha diversity was significantly decreased in the model rats compared to the normal rats (*P* < 0.05) and increased with SMY treatment (*P* < 0.01). The most abundant phyla were *Firmicutes* and *Bacteroidetes*, followed by *Proteobacteria*, *Verrucomicrobia*, and *Actinobacteria*. At the genus level, there was a lower relative abundance of *Lactobacillus*, *Bacteroides*, *Akkermasia*, and *Allobaculum*, and a higher relative abundance of *Lachnospiraceae NK4A 136 group, Ruminococcaceae UCG-014*, and *Sphingomonas* in the MC group*.* The relative abundance of *Actinobacteria*, *Alistipes*, *Bifidobacterium*, *Bifidobacterium*, *Bifidobacteriaceae*, *Lachnospiraceae NK4A136group*, *Lactobacillus*, *Lactobacillaceae*, *Bacilli*, *Verrucomicrobiae*, and *Akkermansia* were significantly abundant in the treatment groups, and thus may be singled out as potential biomarkers for SMY in the treatment of spleen deficiency. In addition, analysis on the correlation between species and physicochemical indexes showed that the abundance of *Parasutterella* was negatively correlated with the change in GAS, and positively correlated with the change in VIP (*P* < 0.01).

**Conclusion:**

Our findings have provided preliminary evidence that modulating the gut microbiota may play a role in the treatment of spleen deficiency with SMY. However, further studies are needed to clarify the mechanism by which SMY regulation of related gut microbiota occurs.

## Background

In traditional Chinese medicine (TCM), spleen-deficiency syndrome is an important pathological change that is featured by loose stool, increased frequency of bowel movements, persistent diarrhea, less dietary intake, poor appetite, and abdominal bloating [1–5]. An important pathological change in TCM, spleen deficiency syndrome has been correlated with the occurrence and development of a wide range of diseases. For instance, epidemiological research has revealed that 64.04% of functional dyspepsia can be attributed to spleen deficiency [1]. Spleen deficiency is attributable to disturbed D-xylose, gastrin (GAS), and vasoactive intestinal peptide (VIP), and accumulating evidence in recent years is also suggestive of its association with dysbacteriosis [6]. The role of gut microbiota alterations in spleen-deficiency has been receiving increasing attention, and TCM has been considered a safe and effective approach for modulating intestinal microbiota [7]. Shengmai Yin (SMY), a TCM formula first recorded in the Origins of Medicine (Yī Xué *Qĭ Yuán*) by Yuansu Zhang (A.D. 1186), consists of three herbs: *Radix ginseng, Radix ophiopogonis*, and *Fructus schisandrae*. Some of the main effective components of SMY are saponins, polysaccharides, flavonoids, and lignans. It has long been used widely for the cardiovascular diseases of Qi-Yin deficiency syndrome and its cardioprotective actions on multiple pathways have been well documented [8, 9]. At present, the SMY decoction has been developed into a variety of preparations including SMY, SMY capsules, SMY granules, SMY injections, and SMY tablets. SMY first appeared in published literature in the first part of the Chinese pharmacopoeia in 1985. According to statistics, SMY accounts for about 30% of the total sales volume of China's pharmaceutical retail market. SMY is also sold in other countries, including the United States, Europe, Japan, South Korea, among others. Intestinal flora can participate in the transformation of the chemical components of traditional Chinese medicine in vivo. A large number of experiments have proved that the biotransformation of intestinal flora is an influencing factor for improving the bioavailability of saponins, alkaloids, and flavonoids [10, 11]. Interestingly, our previous study revealed the therapeutic effects of SMY and its polysaccharides on rats with diarrhea due to spleen-deficiency [12]. SMY has the effect of tonifying Qi, and Panax ginseng (ginseng) in SMY is well known as a key herb for replenishing Qi and tonifying the spleen. It has also been proven that ginseng can ameliorate spleen deficiency [13]. Furthermore, some of its ingredients, such as ginseng polysaccharide, ophiopogon japonicus polysaccharide MDG-1, and schisandra polysaccharide have been proven to modulate the gut microbiota, enrich the diversity of gut microbiota, and increase the proliferation of probiotics [14–16]. Despite this, few studies have explored the effect of SMY on the intestinal microbiota in spleen-deficiency.

Hereby, to testify the relationship between the gut microbiota and SMY, we continued our research using a high-throughput sequencing technique to determine changes in the microbial community structure in the intestines using a spleen-deficiency rat model and then further analyzed differences in the gut microbiota between normal rats, spleen-deficiency rats, and SMY-intervened rats.

## Materials and methods

### Chemicals and reagents

*Ginseng Rubra Radix et Rhizoma* (Batch No. 1602008), *Ophiopogonis Radix* (Batch No. 1604042), and *Schisandrae Chinensis Fructus* (Batch No. 1606080) were purchased from Sichuan Neautus TCM Co., Ltd. and identified by two herbology professors (Chengdu, China).

### Experimental animals

A total of 24 Sprague–Dawley 7-day-old male rats weighing 200–220 g were provided by the DaShuo Biotechnology Co., Ltd, [IACUC number: SCXK (Sichuan) 2015-030, Chengdu, China]. The rodents were raised in the specific-pathogen free animal center of Chengdu University of Traditional Chinese Medicine (CDUTCM) at 22 ± 2 °C and 50 ± 10% relative humidity with a 12 h light/dark cycle and free access to water and rodent chow ad libitum. All the animals were fed under the above conditions for one week prior to the experiment. All experiments were performed in line with the Regulations of Experimental Animal Administration and the protocol was approved by the Animal Care and Use Committee of CDUTCM [protocol number: 2015-0082].

### Preparation of *Rheum officinale* and SMY decoction

The preparation and quality control of *Rhei Radix et Rhizoma*, SMY decoction were performed as previously described [12]. In brief, the *Rhei Radix et Rhizoma* sample was prepared as a 200% decoction (1 mL rhubarb decoction was equivalent to 2 g of crude material medica). To prepare the SMY decoction, *Ginseng Rubra Radix et Rhizoma*, *Ophiopogonis Radix*, and *Schisandrae Chinensis Fructu*s at a ratio of 1:2:1 were immersed into an eightfold quantity of water at room temperature for 30 min and then decocted for 30 min, twice. The acquired solution was filtered and concentrated to 700 g L^−1^.

### Ultrahigh performance liquid chromatography quadrupole orbitrap high resolution mass spectrometry (UPLC-Q-Orbitrap HRMS)

The SMY decoction was analyzed by ultrahigh performance liquid chromatography quadrupole Orbitrap high resolution mass spectrometry (UPLC-Q-Orbitrap HRMS) (Thermo Fisher Scientific, USA) with an Accucore C chromatographic column (3 mm × 100 mm, 2.6 μm, Thermo Fisher Scientific, USA). The mobile phase consisted of (A) 100% water with 0.1% formic acid and (B) 100% acetonitrile. Gradient elution was conducted using 5%-15% (0–10 min), 15% ~ 18% (10 ~ 18 min), 18% ~ 22% (18 ~ 20 min), 22% ~ 25% (20 ~ 35 min), 25% ~ 30% (35 ~ 45 min), and 30% ~ 40% acetonitrile (45 ~ 60 min). The flow rate was 0.3 mL/min. High resolution mass detection was performed on a Q-Exactive Orbitrap tandem mass spectrometer (Thermo Fisher Scientific, USA) equipped with a heated electrospray ionization source (ESI) operated in the positive and negative ion mode. The ESI source parameters were set as follows: ion spray voltage at 3 kV (±), probe heater temperature at 350 ℃, sheath gas flow rate at 35 arbitrary units, the auxiliary gas flow rate at 10 arbitrary units, and the ion transfer tube temperature at 320 ℃. Full mass spectra were obtained from m/z 100 to 1500 with a resolution of 70,000, while data dependent MS2 (dd-MS2) spectra were acquired at a resolution of 35,000 with the ramp collision energy at 20, 40, and 60 eV.

### Establishment of the Spleen-deficiency rat model

All rats were randomly divided into three groups (n = 8): normal control group (NC), model control group (MC), and Shengmai Yin group (SMY). The spleen-deficiency rat models were established by gastric gavage with 10 mL kg^−1^ of 200% *Rhei Radix et Rhizoma* decoction once a day for 15 consecutive days as previously described by Shen [17], while NC group used 0.9% saline solution instead. The weight and clinical demonstration of all rats were monitored daily.

### Treatment groups

After successful spleen-deficiency modeling, the rodents in each group received the relevant treatments via gastric gavage once a day for 10 consecutive days, namely SMY decoction at a concentration of 10 mL kg^−1^ for the SMY group and an equal volume of physiological saline for the NC and MC groups, respectively. The weight and clinical demonstration of all rats were monitored daily.

### Preparation of samples

On the first day before intervention, blood was sampled from the orbit for the determination of d-xylose. One hour after the treatment on the 10th day, the rodents were weighed and anesthetized with 2% chloral hydrate. Blood was collected from the abdominal aorta, allowed to settle for 1 h at room temperature, and then centrifuged at 3500 rpm 4 °C for 10 min. The supernatant serum was frozen at − 80 °C for the determination of d-xylose, GAS, and VIP. The fecal samples (5 g fecal matter) were collected from the descending colon (5 cm above the anus and 3 cm in length) and stored at − 80 °C for 16S rRNA sequencing. Colon tissue samples were rinsed with isotonic saline and subsequently used to assess colonic mucosa damage.

### Histology analysis

The colonics were washed with saline and immediately fixed in a 12% formaldehyde solution for 24 h. The tissue samples were dehydrated, embedded in paraffin, and sliced using automatic dehydrator. The 4–5 μm sliced tissues were stained with hematoxylin and eosin (HE) and were observed with a BA200 Digital Microscope (Leica, Germany). Images were collected from the selected areas at 100 times and 400 times magnification.

### Resorcinol method and Enzyme-linked immunosorbent assay

The levels of d-xylose [18] in the serum were determined by the resorcinol method using commercially available kits (Nanjing Jiancheng Institute of Bioengineering). The OD was measured with an ultraviolet–visible spectrophotometer at 554 nm. The levels of GAS and VIP [19, 20] were determined by enzyme-linked immunosorbent assay using commercially available kits (Nanjing Jiancheng Institute of Bioengineering) according to the manufacturer’s instructions. The absorbance was measured at 450 nm using a microplate reader. The serum GAS and VIP contents per group were determined using the standard curve. The results were compared with a standard curve constructed with titrating standards.

### Fecal DNA extraction

DNA was extracted from fecal samples using the DNeasy PowerSoil Kit (QIAGEN, Germany) following the instructions of manufacturer. DNA integrity and fragment size range were assessed by agarose gel electrophoresis, and DNA concentrations and quality were measured using a NanoDrop Spectrophotometer 2000C (Thermo Fisher Scientific, USA). DNA was diluted to 10 ng/μL using sterile ultrapure water and stored at − 80 °C for downstream use.

### Illumina MiSeq sequencing

Specific 16S r RNA gene primers 16S-V4: 515F (5′-GTGYCAGCMGCCGCGGTAA-3′) and 806R (5′-GGACTACHVGGGTWTCTAAT-3′) were used. On the 5′ with 12 nt unique barcode. The PCR mixture (25 μL) contained 1× PCR buffer, 1.5 mM MgCl_2_, each deoxynucleoside triphosphate at 0.4 μm, each primer at 1.0 μm, 0.5 U of KOD-Plus-Neo (TOYOBO), and 10 ng template DNA. The PCR amplification program consisted of the initial denaturation at 94 ℃ for 1 min, followed by 30 cycles of denaturation at 94℃ for 20 s, annealing at 54 ℃ for 30 s, and elongation at 72 ℃ for 30 s, with a final extension at 72℃ for 5 min. Three replicates of the PCR reactions for each sample were combined. PCR products were mixed with 1/6 volume of 6× loading buffer and loaded onto 2% agarose gels for detection. Samples with a bright main strip at 300 bp were chosen for further experiments. The electrophoresis band was purified using the OMEGA Gel Extraction Kit (Omega Bio-Tek, USA). DNA was quantified using Qubit@ 2.0 Fluorometer (Thermo Scientific). PCR products from different samples were pooled at equal molar amounts. Sequencing libraries were generated using the TruSeq DNA PCR-Free Sample Prep Kit following manufacturer’s recommendations, and index codes were added. The library quality was assessed on the Qubit@ 2.0 Fluorometer (Thermo Scientific) and the Agilent Bioanalyzer 2100 system. At last, the library was applied to paired-end sequencing (2 × 250 bp) with the Illumina Hiseqapparatus at Rhonin (Biosciences Co., Ltd).

### Statistical analysis

Data were expressed as mean ± SD for normally distributed data, and as M (Q_25_–Q_75_) for non-normally distributed data. One-way analysis of variance (ANOVA) was performed for normally distributed data, and a non-parametric test (Kruskal–Wallis *H* test.) was used for non-normally distributed data. Differences in the relative abundances between the groups were assessed using the Kruskal–Wallis test. Bioinformatic analyses were performed using R3.2.3 (https://cran.r-project.org). Alpha diversity was calculated using Simpson’s diversity index. Beta diversity was determined by analysis of similarities (ANOSIM) using unweighted UniFrac as the distance metric. OTUs that were differentially abundant were determined by Machine Learning (Random Forest). Results were deemed significant upon *P* < 0.05.

## Results

### Chemical components of SMY

**Fig. 1:**
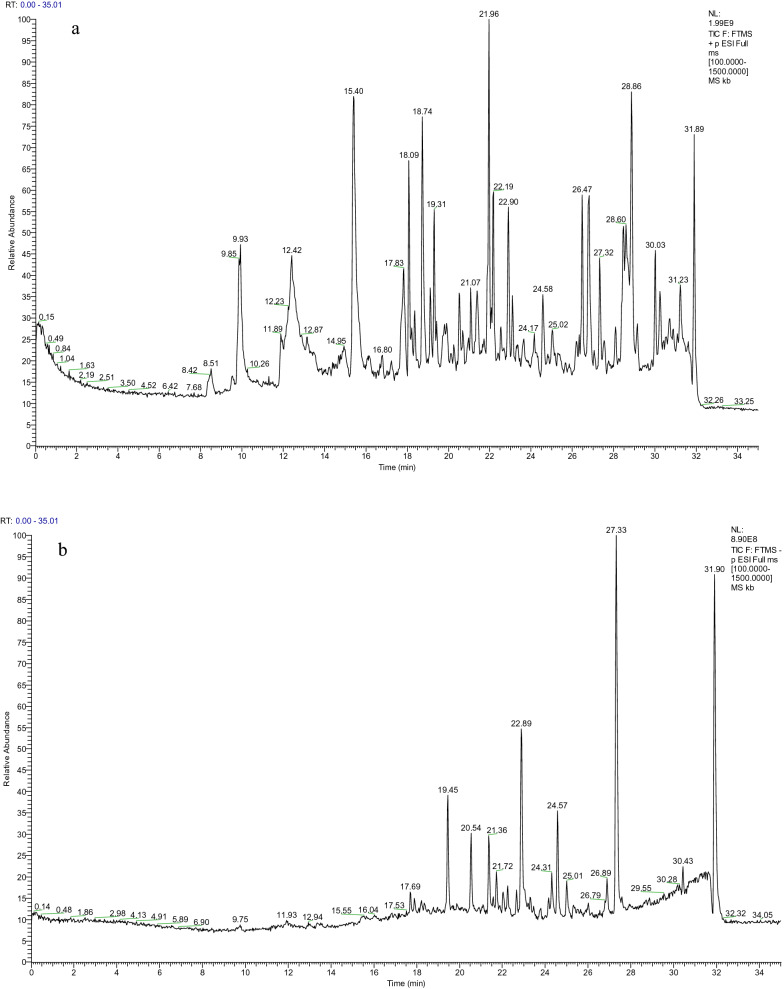
Total ions chromatograms of SMY in positive (**a**) and negative (**b**) ion modes. Identification of chemical components of SMY was listed in Table 1

We analyzed the main components of SMY using UPLC-Q-Orbitrap HRMS and successfully identified the potential main components of SMY, that is, Ginsenoside Rg2, GinsenosideRh1, GinsenosideRg3, Notoginsenoside R2, Ruscogenin, Schizandrin A, Gomisin D, Schisanhenol, and Methylophiopogonanone A (Fig. 1, Table 1).

**Table 1:** Chemical components of SMY

Number	*t*_R_/min	Ion peak	*δ*/ppm	Molecular formula	Fragment ion	Compound
1	1.36	191.05537[M−H]^−^	3.84	C_7_ H_12_ O_6_	127.03906,93.03358,85.02847	D-(−)-Quinic acid
2	1.46	173.04488[M−H]^−^	3.82	C_7_ H_10_ O_5_	137.02368,111.00795,111.04430,93.03369,85.02858	Shikimic acid
3	2.41	143.03433[M+H]^+^	− 2.19	C_6_ H_6_ O_4_	125.02372,97.02904	cis,cis-Muconic acid
4	2.77	169.01361[M−H]^−^	3.74	C_7_ H_6_ O_5_	125.02351	Gallic acid
5	3.95	154.05022[M+H]^+^	− 1.87	C_7_ H_7_ N O_2_	126.05517	3-Aminosalicylic acid
6	4.50	220.11823[M+H]^+^	− 0.96	C_9_ H_17_ N O_5_	202.10765,184.09728,124.07614,116.03467,90.05563	Pantothenic acid
7	5.03	113.06023[M+H]^+^	− 4.58	C_6_ H_8_ O_2_	95.04972,85.06541	Sorbic acid
8	5.38	153.01875[M−H]^−^	3.96	C_7_ H_6_ O_4_	123.00771,109.02868,108.02083,95.01298,85.02846	Gentisic acid
9	5.41	173.00856[M−H]^−^	3.63	C_6_ H_6_ O_6_	111.00781	trans-Aconitic acid
10	5.98	337.09299[M−H]^−^	− 0.07	C_16_ H_18_ O_8_	191.05562,163.03938,119.04932	3-p-Coumaroylquinic acid
11	6.03	353.08786[M+H]^+^	− 0.93	C_16_ H_18_ O_9_	191.05598,179.03450,173.04495,135.04433,93.03380	Neochlorogenic acid
12	6.53	353.08832[M−H]^−^	− 0.95	C_16_ H_18_ O_9_	191.05559	Chlorogenic acid
13	6.60	167.03435[M−H]^−^	3.78	C_8_ H_8_ O_4_	152.01073	Vanillic acid
14	7.13	177.01866[M−H]^−^	3.63	C_9_ H_6_ O_4_	133.02861,105.03360,89.03863	Esculetin
15	7.51	179.03439[M−H]^−^	3.28	C_9_ H_8_ O_4_	135.04424	Caffeic acid
16	7.53	140.03442[M+H]^+^	− 1.8	C_6_ H_5_ N O_3_	112.0398	6-Hydroxynicotinic acid
17	7.58	193.0498[M+H]^+^	− 1.57	C_10_ H_8_ O_4_	165.05478,137.05975	5,7-Dihydroxy-4-methylcoumarin
18	7.88	211.13313[M+H]^+^	− 1.09	C_12_ H_18_ O_3_	193.12248,175.11176,151.11180,133.10124,123.08053	Jasmonic acid
19	7.99	153.05502[M−H]^−^	4.51	C_8_ H_10_ O_3_	138.03146,109.02866	Vanillyl alcohol
20	8.25	127.03925[M+H]^+^	− 4.24	C_6_ H_6_ O_3_	109.02896,81.03424	Maltol
21	9.16	121.06519[M+H]^+^	− 3.35	C_8_ H_8_ O	103.05474,93.07050,91.05482	Acetophenone
22	9.23	167.03456[M−H]^−^	3.78	C_8_ H_8_ O_4_	152.01074,111,00,785	Vanillic acid
23	9.62	197.11755[M+H]^+^	− 3.04	C_11_ H_16_ O_3_	179.10684,161.09627,135.11705,133.10144,107.08598	Loliolide
24	9.68	165.05519[M−H]^−^	3.24	C_9_ H_10_ O_3_	147.04446,121.02873,119.04942,72.99212	D( +)-Phenyllactic acid
25	9.82	303.05109[M−H]^−^	− 0.2	C_15_ H_12_ O_7_	285.04037,125.02352	Taxifolin
26	9.94	153.05486[M+H]^+^	− 1.8	C_8_ H_8_ O_3_	135.0442	2-Anisic acid
27	9.97	205.08188[M−H]^−^	− 0.03	C_11_ H_13_ N O_3_	164.07118,147.04456	N-Acetyl-L-phenylalanine
28	10.01	163.03943[M−H]^−^	4.54	C_9_ H_8_ O_3_	119.04939	3-Coumaric acid
29	10.07	193.05038[M−H]^−^	2.53	C_10_ H_10_ O_4_	178.02650,149.00001,134.03650	Ferulic acid
30	10.11	223.06119[M−H]^−^	0.18	C_11_ H_12_ O_5_	208.03734,193.01373,164.04703,149.02371,121.02860	Sinapinic acid
31	10.54	197.11781[M+H]^+^	− 2.83	C_11_ H_16_ O_3_	179.10692,161.09637,135.11708,133.10147,	Loliolide
32	10.78	193.05014[M−H]^−^	0.34	C_10_ H_10_ O_4_	178.02655,134.03648	Ferulic acid
33	10.80	221.19048[M+H]^+^	− 0.81	C_15_ H_24_ O	203.17960,147.11691,119.08584,109.10159,105.07030	(-)-Caryophyllene oxide
34	10.84	225.07610[M+H]^+^	− 1.36	C_11_ H_12_ O_5_	207.06557,175.03934,147.04431,119.04958,91.05489	Sinapinic acid
35	11.62	609.14636[M−H]^−^	− 0.83	C_27_ H_30_ O_16_	301.03519,300.02737,271.02457,243.02968	Rutin
36	11.64	463.08810[M−H]^−^	− 0.59	C_21_ H_20_ O_12_	301.03540,300.02747,271.02469,255.02977,243.02968	Quercetin-3β-D-glucoside
37	12.03	433.07727[M−H]^−^	− 0.86	C_20_ H_18_ O_11_	301.03549,300.02722,271.02472,255.02969,243.02960	quercetin-3-O-pentoside
38	12.10	137.13268[M+H]^+^	− 0.84	C_10_ H_18_ O	95.08604,81.07051	Eucalyptol
39	12.48	287.09183[M+H]^+^	− 1.25	C_16_ H_14_ O_5_	167.03418	Sakuranetin
40	12.72	265.14398[M+H]^+^	− 1.26	C_15_ H_20_ O_4_	247.13326,209.08113,163.07553,135.08072,107.08610	( ±)-Abscisic acid
41	12.80	447.09378[M−H]^−^	− 1.93	C_21_ H_20_ O_11_	285.04077,284.03268,255.02979,227.03462	Astragalin
42	13.33	193.04997[M−H]^−^	2.53	C_10_ H_10_ O_4_	178.02667,149.06006,134.03658	Ferulic acid
43	13.36	253.17989[M+H]^+^	− 0.31	C_15_ H_24_ O_3_	235.16960,127.07537,99.08104,85.06549,81.07063	(4R,4aS,8aS)-4-Hydroxy-4-(hydroxymethyl)-3,4a,8,8-tetramethyl-4a,5,6,7,8,8a-hexahydro-1(4H)-naphthalenone
44	13.88	191.03427[M−H]^−^	3.7	C_10_ H_8_ O_4_	147.04442,102.94783	5,7-Dihydroxy-4-methylcoumarin
45	14.74	271.06119[M−H]^−^	0.06	C_15_ H_12_ O_5_	151.00288,119.04927,107.01289,93.03358,83.01283	Naringenin
46	16.05	209.11769[M+H]^+^	− 2.24	C_12_ H_16_ O_3_	194.09410,181.08620,178.09917,168.07838,121.06514	β-Asarone
47	16.45	237.18507[M+H]^+^	− 1.56	C_15_ H_24_ O_2_	219.17474	Dihydroartemisinic acid
48	16.64	285.07699[M−H]^−^	0.44	C_16_ H_14_ O_5_	165.01868,119.04929	5,7-Dihydroxy-4-(4-methoxyphenyl)-2-chromanone
49	16.82	237.18541[M+H]^+^	− 1.9	C15 H24 O2	219.17458	Curcumol
50	16.88	433.22235[M+H]^+^	− 0.29	C_24_ H_32_ O_7_	415.21173,384.19327,369.17017,346.14090,338.1512	Schisandrin
51	17.75	327.21774[M−H]^−^	− 0.6	C_18_ H_32_ O_5_	211.13344,111.00777	Corchorifatty acid F
52	18.08	327.21805[M−H]^−^	− 0.6	C_18_ H_32_ O_5_	242.98550,211.13348,183.13852,97.06501,85.02849	Corchorifatty acid F
53	18.37	769.47351[M−H]^−^	1.08	C_41_ H_70_ O_13_	637.43298,475.37967,113.02346,101.02346,71.01279	20(R)-Notoginsenoside R2
54	18.45	343.11826[M+H]^+^	− 1.15	C_19_ H_18_ O_6_	135.04422	Methylophiopogonanone A
55	18.64	531.22351[M+H]^+^	− 1.64	C_28_ H_34_ O_10_	401.15988,383.14908,352.13083,341.10263,337.10757	Gomisin D
56	18.78	783.48883[M−H]^−^	1.07	C_42_ H_72_ O_13_	637.43164,475.37878,391.28574,101.02344,71.01279	20(R)-Ginsenoside Rg2
57	18.90	769.47498[M−H]^−^	1.04	C_41_ H_70_ O_13_	637.43268,475.38022,115.91991,101.02350,71.01286	20(R)-Notoginsenoside R2
58	18.90	501.24854[M+H]^+^	− 0.77	C_28_ H_36_ O_8_	401.19583,370.17758,369.16953,337.14297,323.12762	Angeloylgomisin H
59	18.94	637.43054[M−H]^−^	0.69	C_36_ H_62_ O_9_	637.43323,475.38144,161.04483,113.02383,101.02373	20(R)-Ginsenoside Rh1
60	19.09	783.48932[M−H]^−^	1.07	C_42_ H_72_ O_13_	637.43451,475.38000,391.28564,113.02354,101.02354	20(R)-Ginsenoside Rg2
61	19.19	433.22247[M+H]^+^	− 0.37	C_24_ H_32_ O_7_	415.21188,384.19351,369.16995,353.17542,322.15680	Schisandrin
62	19.34	637.43042[M−H]^−^	0.7	C_36_ H_62_ O_9_	637.43121,475.38043,161.04475,101.02357	20(R)-Ginsenoside Rh1
63	19.44	501.24823[M+H]^+^	0.02	C_28_ H_36_ O_8_	401.19647,370.17783,369.17078,337.14359,323.12793	Angeloylgomisin H
64	19.59	129.17468[M+H]^+^	− 0.72	C_15_ H_22_ O	137.13260,123.11709,95.08604,83.04976,81.07051	Zerumbone
65	19.70	457.36844[M+H]^+^	− 1.24	C_30_ H_48_ O_3_	439.35272,439.17239,203.17969,191.17955,189.16400	Oleanolic acid
66	19.83	237.18520[M+H]^+^	− 2.12	C_15_ H_24_ O_2_	219.17482,201.16435,161.13277,159.11711,119.08590	Curcumol
67	19.85	219.17484[M+H]^+^	− 0.72	C_15_ H_22_ O	201.1.06405,161.13274,159.11716,119.08595,105.07041	Nootkatone
68	20.03	219.17474[M+H]^+^	− 0.72	C_15_ H_22_ O	159.11697,109.10164,95.08614	Nootkatone
69	20.25	293.17603[M−H]^−^	0.38	C_17_ H_26_ O_4_	236.10509,221.15431,220.14641	6-Gingerol
70	20.28	219.17447[M+H]^+^	− 0.72	C_15_ H_22_ O	201.16428,145.10139,135.11708,109.10167,93.07056	Nootkatone
71	20.45	389.19641[M+H]^+^	− 1.68	C_22_ H_28_ O_6_	357.17053,325.14389,288.09998,287.09196,227.07072	3-(5-Hydroxy-2,2,7,8-tetramethyl-6-oxo-7,8-dihydro-2H,6H-pyrano[3,2-g]chromen-10-yl)hexanoic acid
72	20.72	403.21173[M+H]^+^	− 0.46	C_23_ H_30_ O_6_	371.18570,333.13324,302.11505,301.10706,287.09171	Schisanhenol
73	21.12	313.23868[M−H]^−^	− 1.38	C_18_ H_34_ O_4_	295.22781,183.13847,129.09103,99.08048	( ±)12(13)-DiHOME
74	21.22	403.21201[M+H]^+^	− 0.47	C_23_ H_30_ O_6_	371.18558,340.16702,302.11496,287.09161,227.07039	Schisanhenol
75	21.59	341.1026[M−H]^−^	0.13	C_19_ H_18_ O_6_	206.05794,178.06284	Methylophiopogonanone A
76	21.68	315.25345[M+H]^+^	− 0.75	C_18_ H_34_ O_4_	183.13847,129.09116,99.08051	( ±)12(13)-DiHOME
77	22.08	417.22736[M+H]^+^	− 0.49	C_24_ H_32_ O_6_	402.20383,347.14920,316.13083,301.10721,285.11203	Schizandrin A
78	22.10	737.41095[M−H]^−^	0.49	C_39_ H_62_ O_13_	163.06015,119.03417,101.02347,89.02344,71.01279	Polyphyllin VI
79	22.50	413.30502[M+H]^+^	0	C_27_ H_40_ O_3_	395.29483,269.19028,251.17978,157.10143,145.10144	Testosterone cypionate
80	23.22	425.37814[M+H]^+^	0.8	C_30_ H_48_ O	121.10139,109.10157,107.08596,95.08605,81.07049	Lupenone
81	23.44	401.19589[M+H]^+^	− 0.02	C_23_ H_28_ O_6_	300.09918	NCGC00163663-02!
82	23.46	425.37805[M+H]^+^	0.79	C_30_ H_48_ O	135.11716,109.10172,107.08609,95.08617,81.07062	Lupenone
83	24.06	443.38809[M+H]^+^	0.58	C_30_ H_50_ O_2_	425.37762,207.17442,135.11687,95.08601,81.07048	Betulin
84	24.06	425.37738[M+H]^+^	0.78	C_30_ H_48_ O	121.10150,109.10164,107.08601,95.08609.91.05466	Lupenone
85	24.07	783.48846[M−H]^−^	1.57	C_42_ H_72_ O_13_	621.43500,161.04472,113.02341,101.02339,71.01275	20(R)-Ginsenoside Rg3
86	24.26	443.38806[M+H]^+^	0.82	C_30_ H_50_ O_2_	425.37790,207.17436,109.10152,95.08606,81.07048	Betulin
87	24.26	783.48883[M−H]^−^	1.56	C_42_ H_72_ O_13_	113.02341,101.02339,71.01274	20(R)-Ginsenoside Rg3
88	24.70	425.37808[M+H]^+^	0.78	C_30_ H_48_ O	147.11687,107.11687,109.10153,95.08601,81.07049	Lupenone
89	25.09	431.31549[M+H]^+^	0.23	C_27_ H_42_ O_4_	287.20078,269.18994,251.17944,139.07545,121.06513	Ruscogenin
90	25.09	413.30848[M+H]^+^	0.41	C_27_ H_40_ O_3_	269.19000,251.17937,210.14043,145.10138,115.07576	Testosterone cypionate
91	25.40	415.32138[M+H]^+^	− 0.46	C_27_ H_42_ O_3_	271.20599,253.19543,157.10149	Diosgenin
92	25.66	415.3208[M+H]^+^	− 0.46	C_27_ H_42_ O_3_	271.20615,253.19553,157.10158	Diosgenin
93	25.75	425.37756[M+H]^+^	0.8	C_30_ H_48_ O	135.11699,109.10159,107.08597,95.08607,81.07052	Lupenone
94	26.04	425.37726[M+H]^+^	0.79	C_30_ H_48_ O	123.11709,109.10161,107.08597,95.08606,81.07053	Lupenone
95	26.04	443.38800[M+H]^+^	0.62	C_30_ H_50_ O_2_	425.37799,207.17436,189.16388,95.08603,81.07047	Betulin
96	26.11	415.32059[M+H]^+^	− 0.52	C_27_ H_42_ O_3_	271.20575,253.19521,157.10138	Diosgenin
97	26.25	355.28464[M+H]^+^	− 0.22	C_21_ H_38_ O_4_	263.23706,245.22650,109.10155,95.08602,81.07050	1-Linoleoyl glycerol
98	26.96	415.32141[M+H]^+^	− 0.46	C_27_ H_42_ O_3_	271.20563,253.19502,157.10127	Diosgenin
99	27.03	425.37860[M+H]^+^	0.78	C_30_ H_48_ O	105.07032	Lupenone
100	27.64	283.26416[M−H]^−^	0.11	C_18_ H_36_ O_2_	163.11212,107.04922	Stearic acid
101	27.96	338.34177[M+H]^+^	− 0.08	C_22_ H_43_ N O	321.31552,97.10168,83.08614,69.07059	Erucamide
102	30.91	415.32095[M+H]^+^	− 0.79	C_27_ H_42_ O_3_	271.20575,253.19533,157.10135	Diosgenin

### Identification of saponins

**Fig.2:**
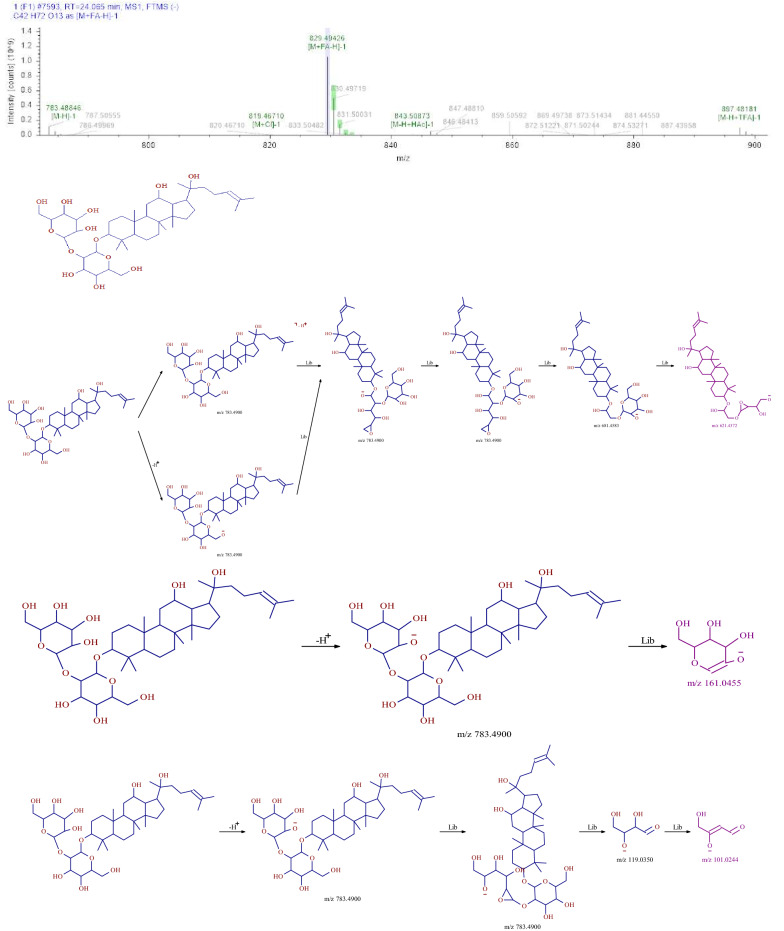
MS^2^ chromatogram and the proposed mass fragmentation patterns of 20 (R)—Ginsenoside Rg3

Six saponins were identified from SMY, including ginsenoside Rg2, ginsenoside Rh1, Ginsenoside Rg3, Notoginsenoside R2, and Ruscogenin, all of which were from ginseng and Ophiopogon japonicus. Using 20 (R)—Ginsenoside Rg3 as an example, the retention time of the compound in the test sample was 24.07 min. Based on the relevant information of the first-order mass spectrometry data, we obtained a molecular ion peak of m/z 783.48846[M−H]^−^. Therefore, the relative molecular weight of the compound was determined to be 784. The original mass spectra data were analyzed and the molecular formula was predicted to be C_42_ H_72_ O_13_. In this experiment, the fragment ions of m/z 621.4372, 161.0455, and 101.0244 corresponded to [M−H-C_4_H_6_O_3_-C_2_H_4_O_2_]^−^, [M−H-C_36_H_62_O_8_]^−^, and [M−H-C_38_H_64_O_9_-H_2_O]^−^, respectively. The compound was finally identified as 20 (R)—Ginsenoside Rg3, based on its fragmentation mode and fragmentation characteristics. The fragmentation pathway of MS is shown in Fig. 2.

### Identification of lignans

**Fig. 3:**
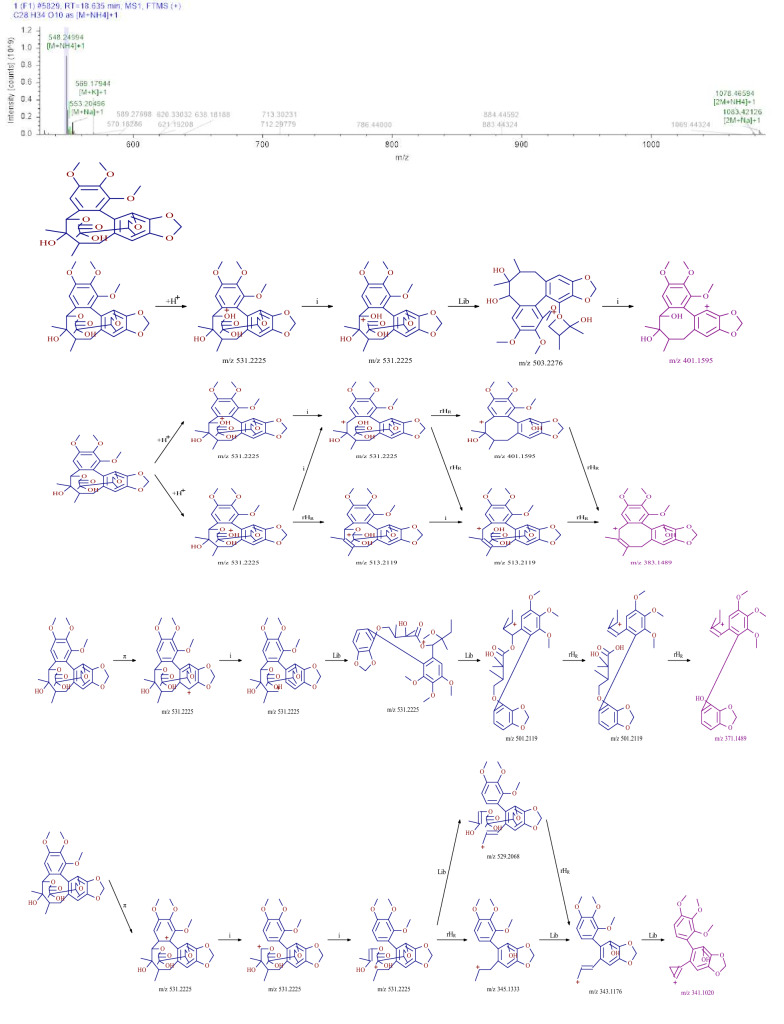
MS^2^ chromatogram and the proposed mass fragmentation patterns of gomisin D

Five lignans were identified from SMY, including schisandrin, schisandrin A, and gomisin D. Using compound gomisin D as an example, the retention time of the compound in SMY was 18.64 min. The first-order mass spectrometry information showed that the positive ion mode was better than the negative ion mode, and the excimer ion peak m/z 531.22351 [M+H]^+^ was obtained in the positive ion mode. The original mass spectra data were analyzed and the molecular formula was predicted to be C_28_H_34_O_10_. The secondary fragment information of the compound mainly included m/z 401.1595 [M+H–CO-C_5_H_10_O_2_] ^+^, m/z 383.1489 [M+H–CO-C_5_H_10_O_2_]^+^, m/z 371.1489 [M+H-CH_2_O-C_6_H_10_O_3_]^+^, and m/z 341.1020 [M+H-C_9_H_14_O_4_-H_2_-H_2_]^+^. According to the fragmentation characteristics of the compound and using a database search, the compound was identified as gomisin D. The fragmentation pathway of the mass spectrometry is shown in Fig. 3.

### Identification of flavonoids

**Fig.4:**
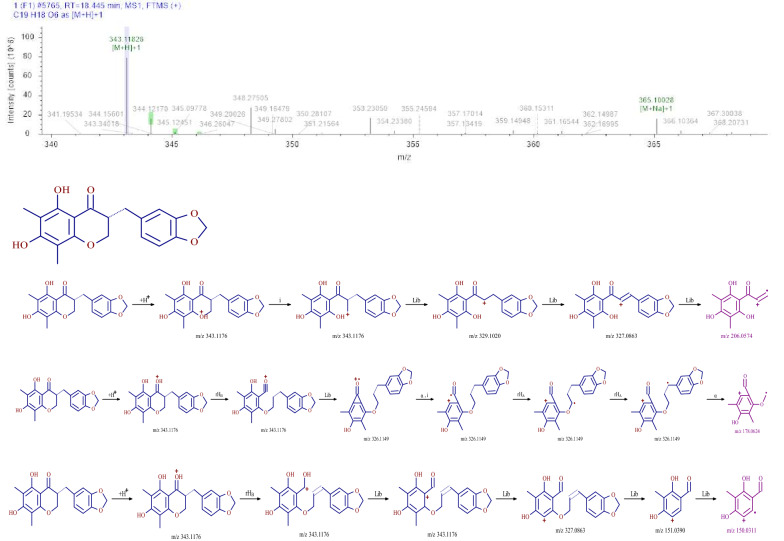
MS^2^ chromatogram and the proposed mass fragmentation patterns of methylophiopogonanone A

In this study, flavonoids were identified from SMY, and included methylophiopogonanone A. With methylophiopogonanone A as an example, the retention time of the compound in SMY was 18.45 min. The first-order mass spectrometry information showed that the response in the positive ion mode was better than that in the negative ion mode, and the excimer ion peak m/z 343.1026 [M+H]^+^ was obtained in the positive ion mode. The original mass spectra data were analyzed and the molecular formula was predicted to be C_19_H_18_O_6_. The secondary fragment information of the compound was mainly m/z 206.0574 [M+H-CH_2_-H_2_-C_7_O_2_H_5_]^+^, m/z 178.0624 [M+H-OH-C_9_H_8_ O_2_]^+^, and m/z 150.0311 [M+H-CH_4_-C_10_ H_8_O_3_]^+^. According to the fragmentation characteristics of the compound and using a database search, the compound was identified as methylophiopogonanone A. The fragmentation pathway of the mass spectrometry is shown in Fig. 4.

### Establishment of the spleen-deficiency rat model

After the gastric gavage of the *Rhei Radix et Rhizoma* decoction, clinical observation showed the rats in the MC and SMY groups started to present different degrees of diarrhea, reduced dietary intake, weight loss, withered fur, fatigue, curling up, arched back, and various degrees of rectocele. From day 3 to 15, the rats in the model group and pre-treatment groups presented persistent diarrhea, whereas clinical manifestation of diarrhea was absent in the NC group. This indicated the successful induction of the spleen-deficiency model.

### Weight and clinical observation index

The rats in the normal control group presented good mental state, quick action, normal dietary intake, and glossy fur. The rats in the model group had diarrhea, reduced dietary intake, mental fatigue, arched back, weight loss, withered fur, and various degrees of rectocele. Compared with the model group, the SMY group showed better improvement. As shown in Table 2, the weights of rodents in the spleen-deficiency MC group decreased significantly compared with those of the normal rats (*P* < 0.01), however, the weights increased significantly in the spleen-deficiency model rats after SMY treatment (*P* < 0.01). The above-mentioned clinical observation indexes of the SMY group were also significantly improved when compared with the MC group.

**Table 2:** Effects of SMY on body weight of spleen-deficiency model rats

Group	Pre-modeling, g	After modeling, g	After the last administration,g
SMY	263.38 ± 9.21	217.00 ± 9.56**	232.63 ± 11.04**^##^
MC	261.88 ± 9.63	185.13 ± 16.44**	204.13 ± 18.63**
NC	259.63 ± 4.66	261.88 ± 11.38	279.63 ± 14.44^##^

vs. NC group, **P < 0.01; vs. MC group, ^##^*P* < 0.01.

### Histological analysis

**Fig. 5:**
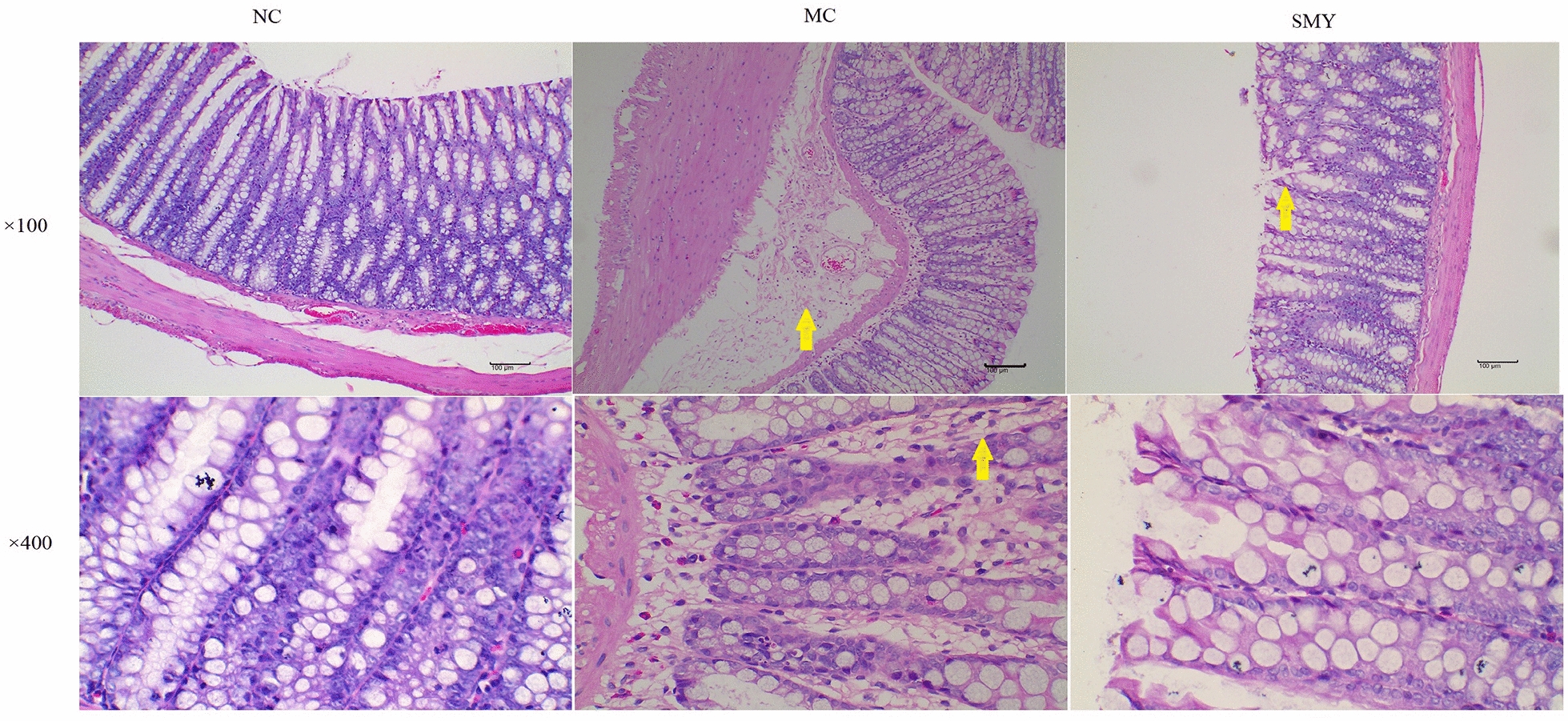
Histopathological observation of rat colonic tissue in different groups (×100 and ×400 magnification). The yellow arrows in the MC colonic sections (×100 and 400 × magnification) indicate edema. The yellow arrows in the SMY colonic sections (×100 magnification) indicate a few exfoliated epithelial cells. There were no significant differences in the histological features between the NC and SMY groups

As shown in Fig. 5, different degrees of lymphocyte infiltration and aggression were observed in the submucosa of spleen-deficiency model rats. No notable congestion, edema, ulcers, inflammatory cell infiltration, or other pathological changes were observed after treatment, and there were no significant differences among the NC and SMY groups.

### Serum levels of D-xylose, GAS, and VIP

D-Xylose is a pentose that is absorbed by the small intestine after oral administration. Under normal circumstances, D-xylose is almost nonexistent in the blood. Therefore, the detection of D-xylose content in the blood after taking a certain dose of D-xylose solution can be used to indirectly evaluate the absorption function of the intestinal mucosa. GAS is a very important gastrointestinal hormone, mainly secreted by the G cells in the gastric antrum, which is an important index for measuring the physiological function of the gastrointestinal tract. VIP is one of the main inhibitory neurotransmitters in the intestine, and can inhibit the secretion of gastric acid. It is an important index of gastrointestinal disease research.

**Fig. 6:**
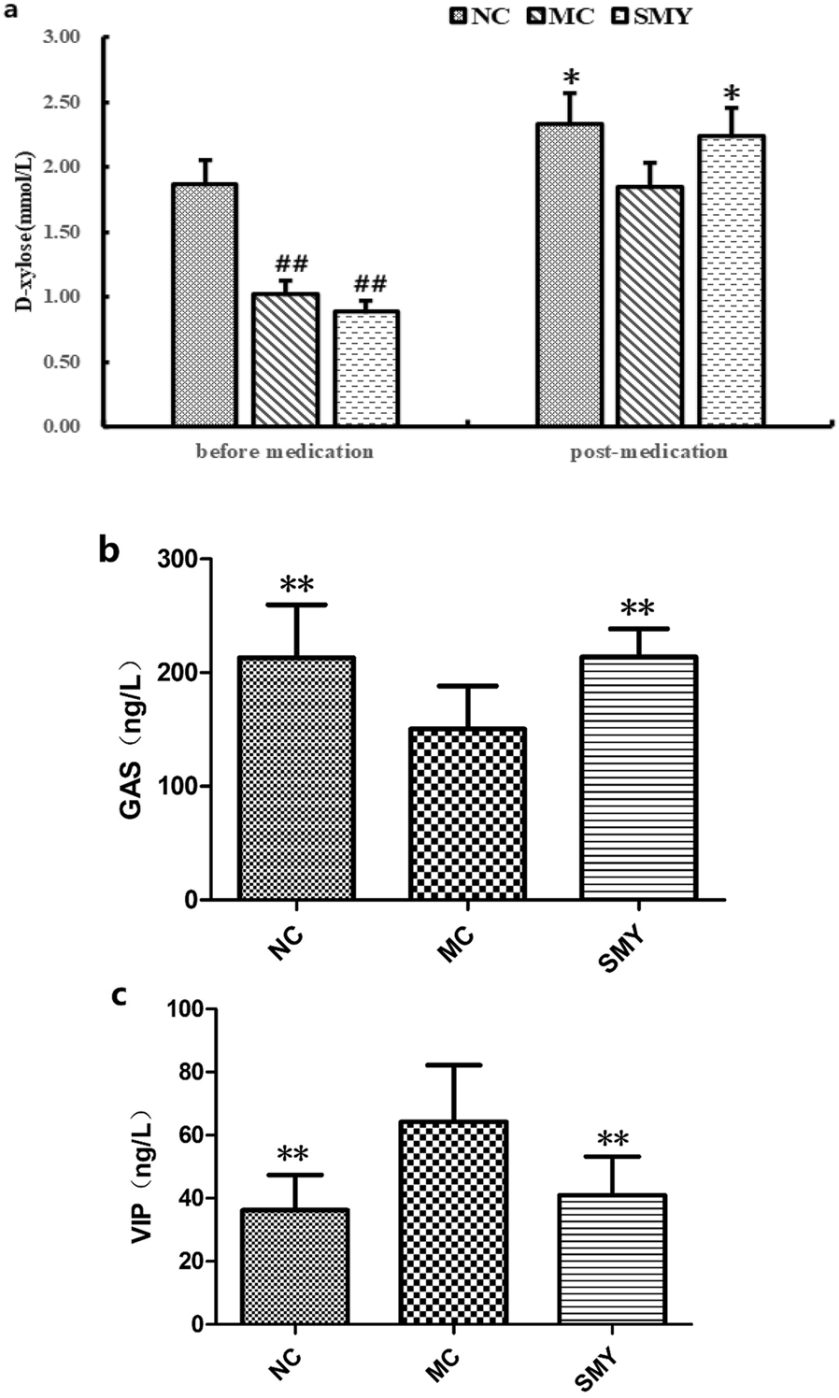
**a** Effects of SMY on the serum content of D-xylose in spleen-deficiency model rats ($\stackrel{-}{\mathrm{x}}$±s, n = 8). Before medication, compared with NC, the serum levels of D-xylose in the other three groups were significantly reduced (^##^*P* < 0.01). After the last administration, SMY increased the serum level of D-xylose (Compare with MC, *P < 0.05). **b** Effects of SMY on the serum content of GAS in spleen-deficiency model rats ($\stackrel{-}{\mathrm{x}}$±s, n = 8). Compared with MC, NC and SMY had increased serum levels of GAS (**P < 0.01). **c** Effects of SMY on the serum content of VIP in spleen-deficiency model rats ($\stackrel{-}{\mathrm{x}}$±s, n = 8). Compared with MC, NC and SMY had reduced serum levels of VIP (**P < 0.01)

As shown in Fig. 6, before medication, the serum levels of d-xylose in the MC and SMY groups were significantly reduced compared with the NC group (*P* < 0.01). After the last administration, the serum levels of D-xylose and GAS were significant increased by SMY (*P* < 0.05, *P* < 0.01), and those of VIP were reduced in SMY group (*P* < 0.01) compared with the MC group.

### Comparison of gut microbial composition in the different groups

**Fig. 7:**
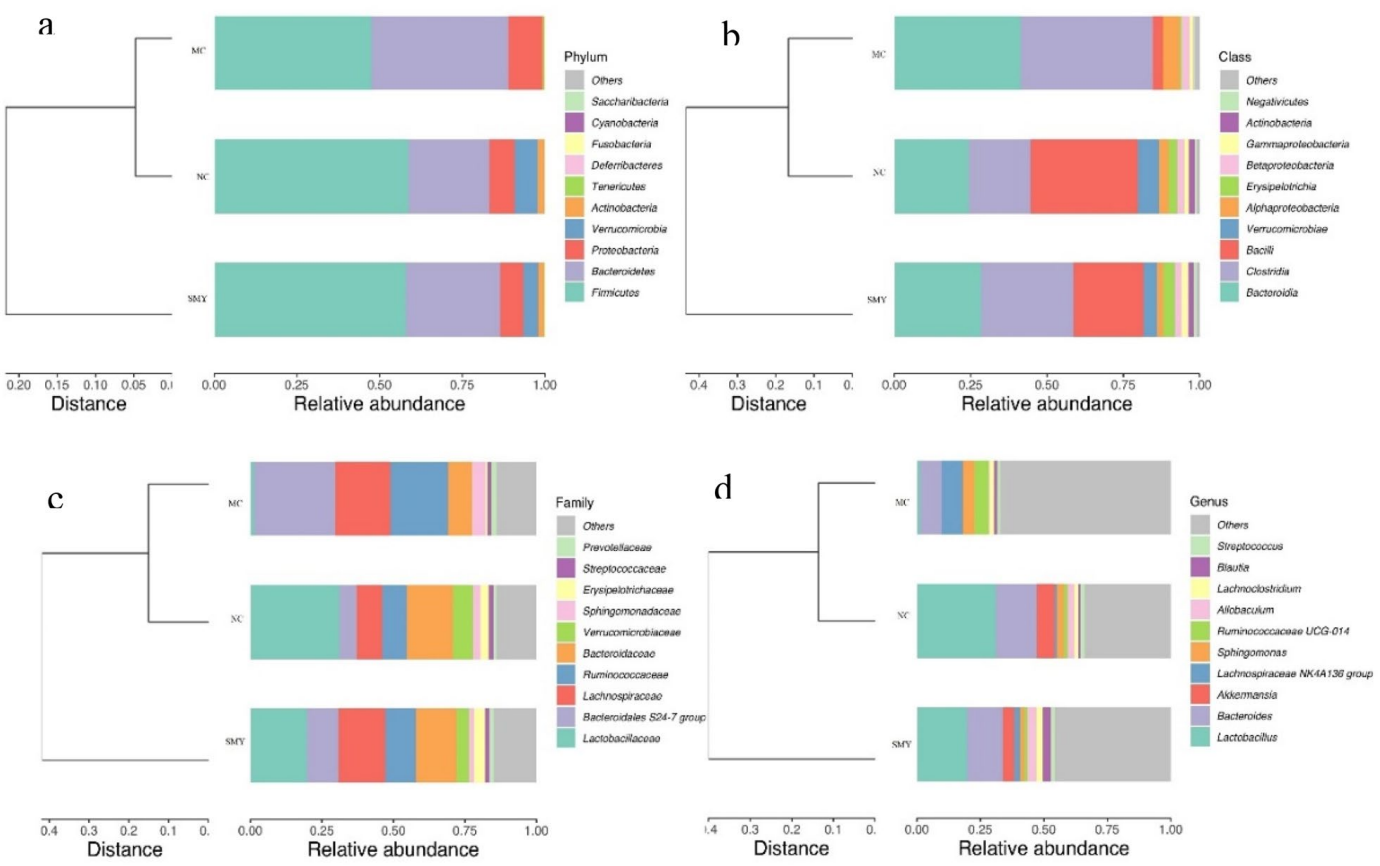
Comparison of community structure at the phylum, class, and genus levels between the groups. **a** At the phylum level; **b** At the class level; **c** At the genus level; **d** Clustering heat map at the genus level

As shown in Fig. 7a, the dominant phyla presented across all groups were *Firmicutes* and *Bacteroidetes*, followed by *Proteobacteria*, *Verrucomicrobia*, and *Actinobacteria*. The fecal samples of the rodents in the NC and MC groups were dominated by *Firmicutes* and *Bacteroidetes*, while the model group had a lower relative abundance of *Firmicutes* and a higher relative abundance of *Bacteroidetes* and *Proteobacteria* compared with the normal rat group. However, at the family level (Fig. 7c), there were some differences among the three groups. For instance, compared with the NC group, the MC group showed an increased abundance of *Bacteroidales S24-7 group*, *Lachnospiraceae,* and *Ruminococcaceae*, while *Lactobacillaceae, Bacteroidaceae,* and *Verrucomicrobiaceae* were decreased. After administration of SMY, the abundance of *Bacteroidales S24-7 group, Lachnospiraceae, Ruminococcaceae, Lactobacillaceae, Bacteroidaceae*, and *Verrucomicrobiaceae* tended to recover to normal levels. Figure 7b and d display the major taxa at the class level across all groups, which were *Clostridia* and *Bacteroidia*, followed by *Bacilli*, *Alphaproteobacteria*, and *Betaproteobacteria*. At the genus level, there was a lower relative abundance of *Lactobacillus*, *Bacteroides*, *Akkermasia*, and *Allobaculum*, accompanied by a higher relative abundance of *Lachnospiraceae NK4A 136 group, Ruminococcaceae UCG-014,* and *Sphingomonas* in the MC group when compared with the normal rats*.* These microbiota profile changes were reversed by the herbal treatment and there were marked differences at both the phylum and genus levels observed among the MC and SMY groups. Of particular note were the observations of the enriching effects on *Firmicutes*, *Actinobacteria*, and *Verrucomicrobia* and the inhibitory effects on *Bacteroidetes* and *Proteobacteria* in the SMY group at the phylum level. Additionally, in the SMY groups at the genus level, the upregulating effects on *Lactobacillus*, *Bacteroides, Akkermansia, Lachnoclostridium*, *Allobaculum, Blautia*, and *Escherichia-Shigella* as well as the downregulating effects on *Lachnospiraceae NK4A 136 group, Sphingomonas*, and *Ruminococcaceae UCG-014* were significantly noted.

### Richness and diversity of gut bacterial communities

**Fig. 8:**
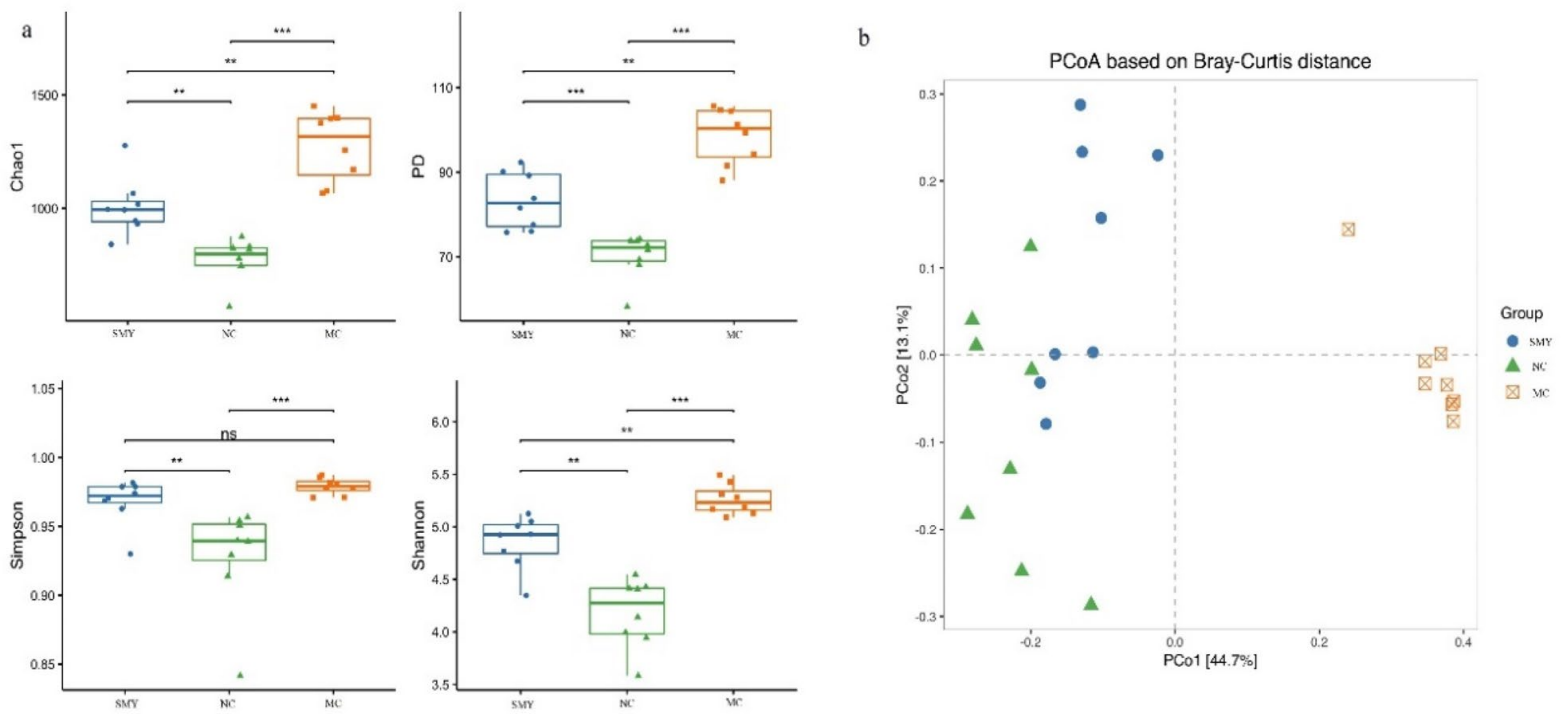
Richness and diversity of gut bacterial communities. **a** Alpha diversity of gut microbiota between the groups. Alpha diversity decreased in the MC rats compared with normal rats, yet significantly increased in the SMY rats compared with spleen-deficiency rats. **b** Beta diversity of gut microbiota between the groups

As shown in Fig. 8a, alpha diversity was significantly decreased in the model rats compared with that of normal rats (*P* < 0.05), which then increased with SMY treatment (*P* < 0.01). In terms of beta diversity, shown in Fig. 8b, the presence of significant differences between the NC and MC groups were demonstrated on the second axis of the principal coordinates analysis (PCoA), indicating that spleen-deficiency may be the factor that attributed to the microbial dysbiosis. The first and second principal coordinates accounted for 41.10% and 11.30% of the total variations, respectively, and in MC were close to the second axis, indicating that other factors affected the microbial community of the spleen-deficiency rats, whereas the SMY were more similar to the NC group.

### Potential biomarkers of spleen-deficiency and different treatments

**Fig. 9:**
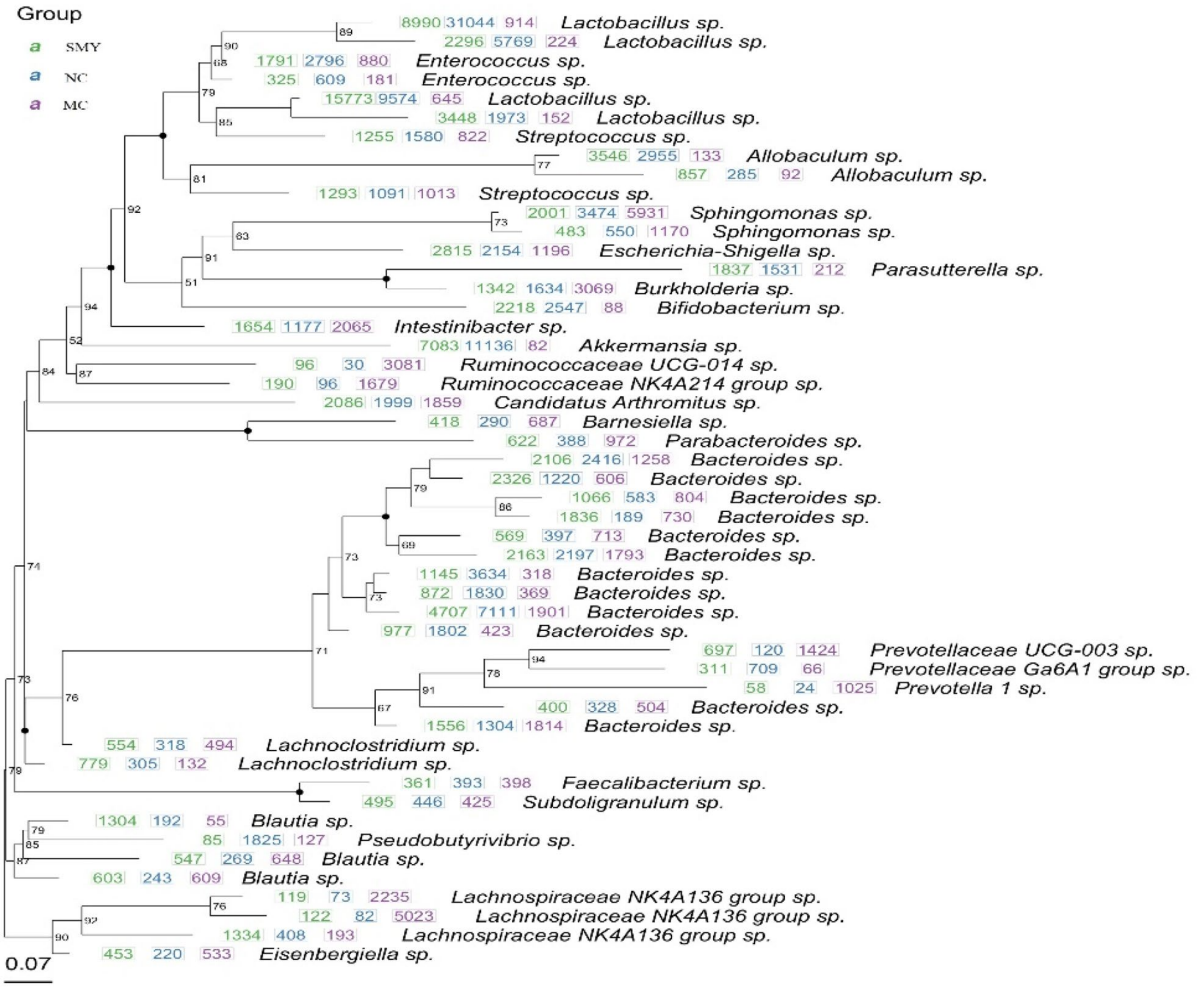
Differently abundant OTUs between the groups

To identify potential biomarkers, random forest was applied to assay which OTUs were differentially abundant among the normal, spleen-deficiency model, and herbal intervention samples. The analysis was performed to infer the contribution of each constituent of the microbiota to the enteric dysbacteriosis of spleen-deficiency rats. The mean decrease in the Gini value identified the most reliable and relevant predictors to perform classifications. A total of 237 OTUs at different taxonomic levels were found to be differentially abundant (*P* < 0.05) between the normal and the model samples; whereas 215 OTUs at different taxonomic levels were differentially abundant (*P* < 0.05) between the MC and herbal treatment groups (Fig. 9).

**Fig. 10:**
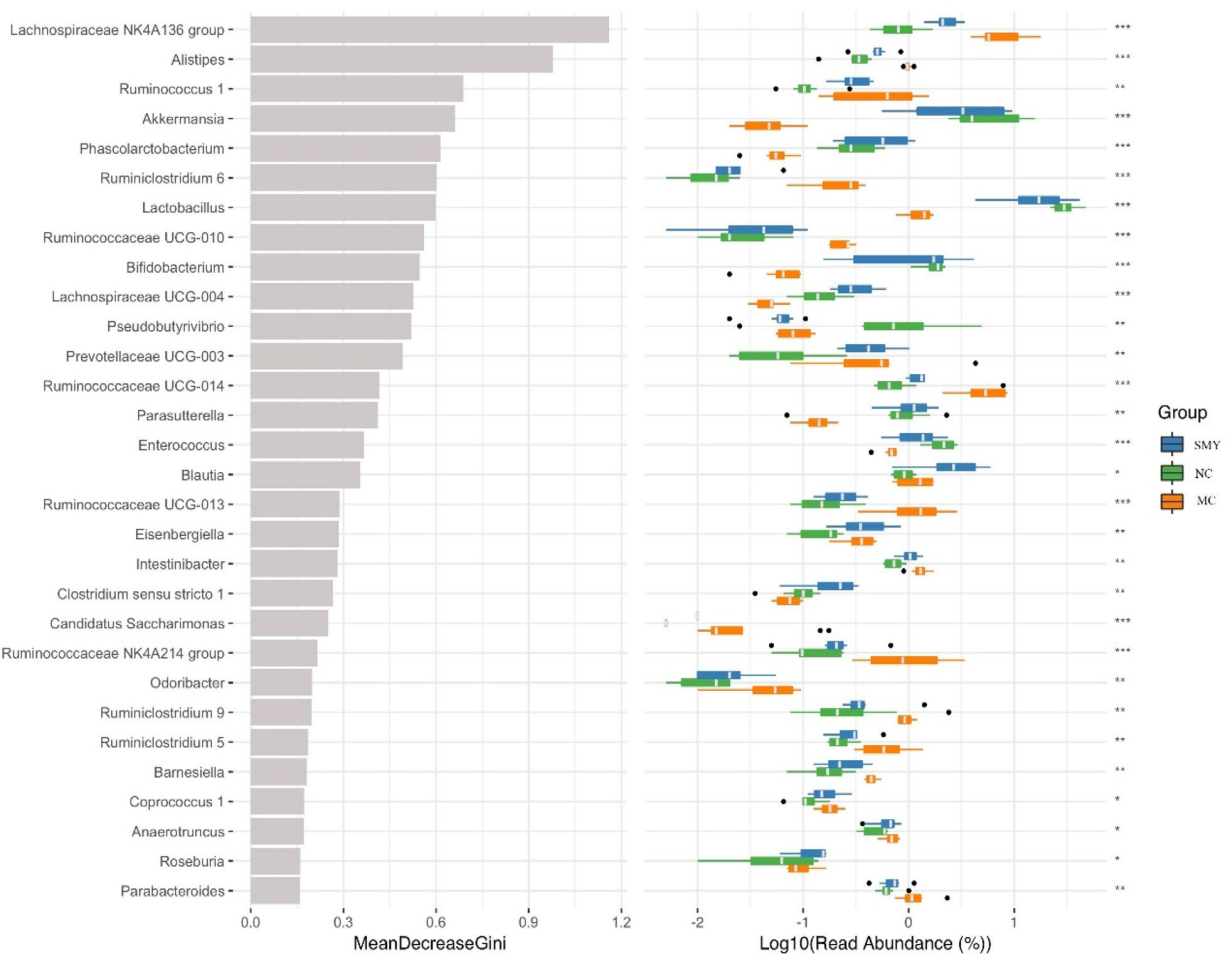
Random Forest analysis between different groups. Blue for the normal group, green model group, orange SMY group. The diameter of each box is proportional to the abundance of taxon. The abscissa on the left is the average decrease of Gini index, the ordinate is the classification information of genera, and the right is the box diagram of the abundance of different groups. The * on the right represents the significance of the difference between groups (Kruskal Wallis rank sum test) (***P < 0.001, **P < 0.01, *P < 0.05). Categories without precise classification information were not analyzed. From the top to the bottom, the importance of influence groups decreases in turn

As shown in Fig. 10, the relative abundances of *Actinobacteria* and *Verrucomicrobia* at the phylum level were highly decreased in the spleen-deficiency model rats; at the class level, *Bacilli*, *Verrucomicrobiae, Erysipelotrichia*, and *Actinobacteria* were decreased, whereas *Clostridia* was enriched in the model rats; at the order level, *Bacteroidales* and *Clostridiales* were significantly enriched, while *Lactobacillales*, *Verrucomicrobiales*, *Erysipelotrichales*, and *Bifidobacteriales* were significantly less abundant in the model rats; at the family level, *Bacteroidales S24-7 group*, *Lachnospiraceae*, *Peptostreptococcaceae*, *Porphyromonadaceae*, *Rikenellaceae*, and *Ruminococcaceae* were enriched in the model rats, while *Lactobacillaceae*, *Verrucomicrobiaceae*, *Erysipelotrichaceae, Enterococcaceae*, and *Bifidobacteriaceae* were enriched in the normal rats. The genera *Lactobacillus*, *Akkermansia*, *Allobaculum*, *Bifidobacterium*, *Enterococcus*, and *Pseudobutyrivibrio* were less abundant, while *Lachnospiraceae NK4A136 group*, *Ruminococcaceae UCG-014*, and *Eubacterium coprostanoligenes group* were more abundant in the spleen-deficiency rodents. Compared with the MC group, the relative abundance of *Actinobacteria*, *Alistipes*, *Bifidobacterium*, *Bifidobacterium*, *Bifidobacteriaceae*, *Lachnospiraceae NK4A136 group*, *Lactobacillus*, *Lactobacillaceae*, *Bacilli*, *Verrucomicrobiae*, and *Akkermansia* were significantly abundant in the herbal intervention groups, which may serve as the potential biomarkers in the SMY based treatment of spleen deficiency.

**Fig. 11:**
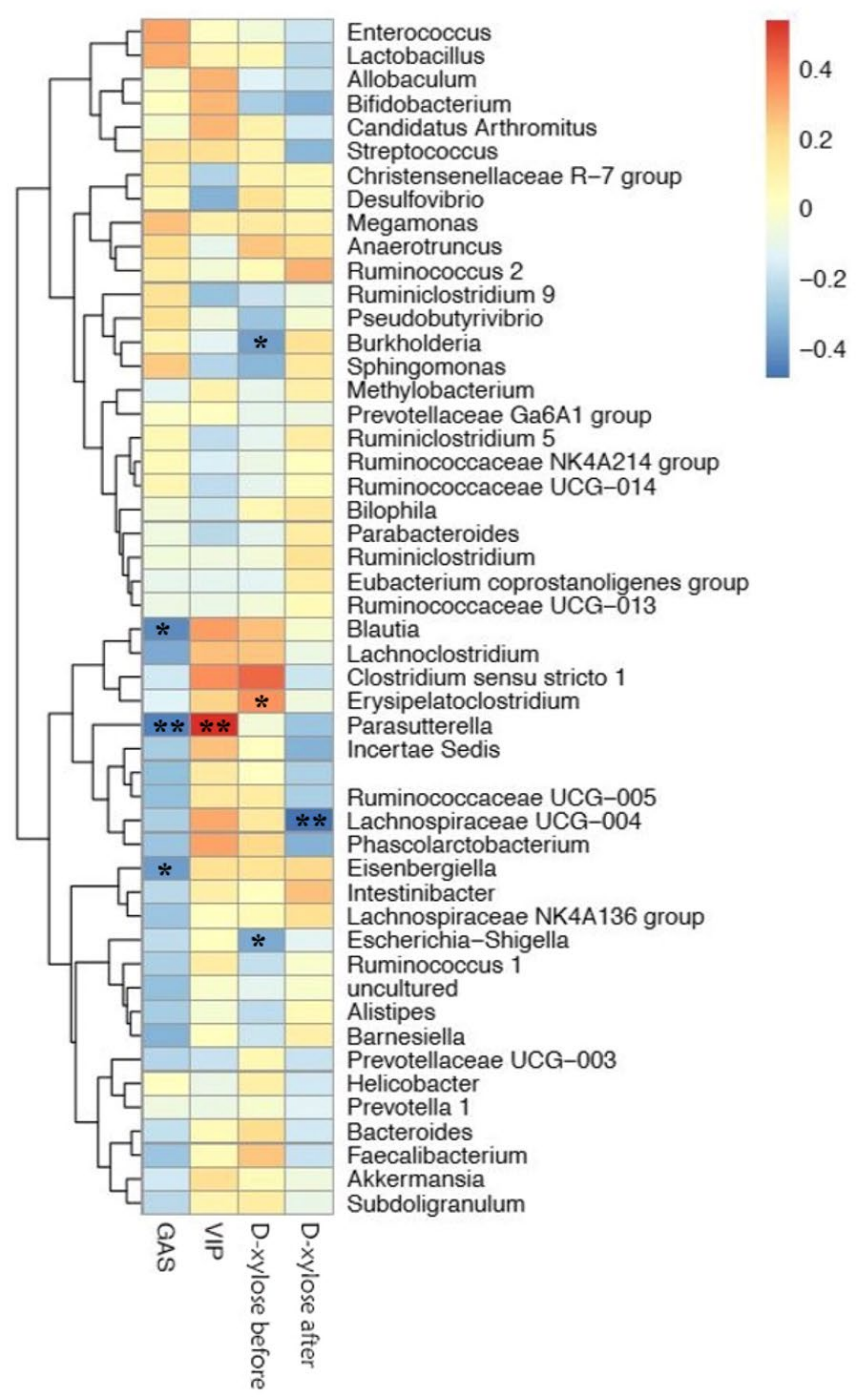
Analysis on the correlation between species and D-xylose, GAS and VIP. The data information of the two-dimensional matrix can be reflected by the color change. The color depth represents the size of the value, and the color gradient can reflect the change trend. The regions in the graph tend to be blue, which represents negative correlation, and red represents positive correlation. The darker the color means the greater the absolute value of correlation coefficient (*P < 0.05, **P < 0.01)

As shown in Fig. 11, further analysis on the correlation between species and physicochemical indexes showed a negative correlation between changes in GAS and the abundance of *Parasutterella* (*P* < 0.01),* Blautia* (*P* < 0.05), and *Eisenbergiella* (*P* < 0.05); VIP was positively correlated with the abundance of *Parasutterella* (*P* < 0.01); and D-xylose was negatively correlated with *Lachnospiraceae UCG-004* (*P* < 0.01), *Burkholderia* (*P* < 0.05), and *Escherichia-Shigella* (*P* < 0.05) (Fig. 11).

## Discussion

In this study, we analyzed the main components of SMY using UPLC-Q-Orbitrap HRMS, and successfully identified the potential main components of SMY (Fig. 1, Table 1), and ginseng Ginseng extracts can significantly increase probiotics in the intestinal flora of rats, including *Bifidobacterium, Lactobacillus, Allobaculum*, *and Clostridium* [21]. It has been found that a water-soluble β-D-fructan polysaccharide from *Ophiopogon japonicas* can increase the number of the intestinal probiotics, especially *Taiwan lactobacillus* and *Lactobacillus murinus* [22]. Although the mechanism of action is not clear, the combination of *Panax ginseng* total saponin and *Atractylodes macrocephala* essential oil significantly ameliorated diarrhea, inhibited intestinal pathology, and modulated gut microbial structure in mice [23]. Due to the tendency for multiple ingredients in Chinese herbal medicines, we only identified the main components of SMY. Further studies are still needed to clarify the main components of SMY and the potential mechanism for the therapeutic effects of SMY on spleen deficiency.

At present, the majority of methods used in the spleen qi deficiency model are aimed at simulating the cause of disease, and include inducing diarrhea with bitter cold [24], improper diet [25], overwork [26], and external dampness trapping spleen [27]. Spleen deficiency syndrome is a pathological condition marked by multiple system dysfunction that is characterized by gastrointestinal digestion hypofunction and gastrointestinal motility disorder [28, 29]. Microecological advances have provided accumulating evidence on the positive correlation between spleen deficiency and alterations in the composition of the gut microbiota [7]. Consistent with previous studies [30, 31], the spleen-deficiency model rats in this study presented with diarrhea, reduced dietary intake, weight loss, different degrees of lymphocyte infiltration and aggression in the submucosa, and a significant reduction in richness and diversity of the gut microbiota. This study further revealed that SMY intervention could reverse the structural variations in the gut microbiota induced by spleen deficiency, and enhance the effects on microbial richness and diversity. The results showed that SMY could improve the alpha diversity in the spleen deficiency model, while SMY treatment showed rats were similar to the NC group in terms of beta diversity, which was significantly different from the spleen deficiency group.

The human gut houses a rich variety of microbes and trillions of gut bacteria have co-evolved with human health [32]. The microbiota possesses a variety of functions and the predominant phyla in the human gut include * Firmicutes*, *Bacteroidetes*, *Actinobacteria*, *Proteobacteria*, and *Verrucomicrobia* [33]. Probiotics (mainly *Bifidobacteria* and *Lactobacilli*) exert actions such as repairing the intestinal mucosal barrier, improving intestinal function, strengthening gut integrity or shaping the intestinal epithelium [34], harvesting energy [35], recovering gastroenteric function, and alleviating gastroenteric symptoms. Additionally, it performs defensive functions and protects against pathogens directly by impeding their colonization through competitive action for space and nutrients or by producing antimicrobial compounds, volatile fatty acids, and chemically modified bile acids [36]. Our results displayed that the rodents with spleen-deficiency had significantly lower abundances of *Firmicutes* and higher abundances of *Bacteroides* and *Proteobacteria* when compared with the normal control group, offering opportunities for conditional pathogen infection. Among the gut microbiome modulated by the herbal intervention, redundancy analysis showed that putative beneficial genera such as *Lactobacillus*, *Bacteroides*, and *Akkermansia* were enriched and that bacterial colonizers or pathogens including *Lachnospiraceae* *NK4A136 group*, *Sphingomonas*, and *Ruminococcaceae* *UCG-014* were reduced as compared with the spleen-deficiency rats. These results further suggested that the therapeutic effect of SMY on spleen deficiency may be attributable to the enrichment of beneficial bacteria and the reduction of pathogenic bacteria.

The primary function of the gut microbiome is to produce short-chain fatty acids (SCFA), mainly including acetate, propionate, and butyrate, during fermentation of undigested resistant starches or oligosaccharides. Spleen deficiency implies a state of low energy metabolism, gastrointestinal digestion hypofunction, and disordered gastrointestinal motility [37–39]. The pathogenic mechanisms involved in spleen deficiency remain unclear, which is partly attributable to the lack of diagnostic and therapeutic biomarkers. In this study, we conducted machine-learning to further uncover the potential biomarkers of spleen deficiency and potential beneficial herbal treatments. Random-forest identified 11 features, and the relative abundance of *Actinobacteria, Alistipes, Bifidobacteriales, Bifidobacterium, Bifidobacteriaceae, Lachnospiraceae NK4A136group, Lactobacillus, Lactobacillaceae, Bacilli, Verrucomicrobiae*, and *Akkermansia* exhibited the highest Gini values, suggesting that these features may be closely related to spleen deficiency and could possibly play a therapeutic role.

Among them, *Ruminococcaceae* and *Lachnospiraceae* are the main butyrate producers in the human gut [40]. Butyrate is a preferred energy source for colonic epithelial cells and is important for maintaining the normal function of the intestinal barrier. The *bifidobacterium* species (Phylum Actinobacteria) produce acetate and lactate during carbohydrate fermentation [41]. Additionally, the mucin-degrading bacteria *Akkermansia muciniphila* (Phylum Verrucomicrobia) produces both propionate and acetate [42]. Lactobacillales have been considered a primary lactic-acid producing bacteria and induce the production of large quantities of anti-inflammatory interleukins that improve intestinal barrier function [43]. The decrease or elimination of lactic acid buildup impairs the intestinal defense barrier and increases osmotic load in the intestinal lumen, ultimately leading to diarrhea [44]. Furthermore, *Alistipes* was found to differ between the healthy SD rats and those rodents with spleen deficiency. Previous studies have shown that a greater frequency of abdominal discomfort is correlated with an increased abundance of several bacterial taxa from the genus *Alistipes* [45]. *Alistipes* may be associated with high frequency of bowel movement or diarrhea in rats with spleen deficiency.

Interestingly, in order to further identify the key intestinal flora involved in regulating spleen deficiency, we correlated the physical and chemical indexes with the intestinal flora. Correlation analysis of the relationship between the genus *Parasutterella* and GAS and VIP revealed that the abundance of *Parasutterella* was correlated negatively with changes in GAS and positively with VIP. Meanwhile, *Parasutterella* that fills the ecological niche in the gastrointestinal tract has been defined as a core component of the human and mouse gut microbiota and contributes to metabolic functionalities [46]. This finding may indicate that *Parasutterella* is a key group of bacteria involved in regulating the spleen deficiency model via SMY. However, further investigation is needed to unravel mechanism by which *Parasutterella* participates in the regulation of spleen deficiency.

Traditionally, SMY is a commonly used formula for cardiovascular diseases [10]. Recent studies have revealed that dysbiosis may elicit the occurrence and development of cardiovascular diseases through interacting with the host’s response to cholesterol metabolism, inflammation, and oxidative stress [47]. In TCM, the spleen, the source of qi and blood generation, has been considered to be closely correlated with the heart, and acts as the imperial guard of the monarch-heart. Our present study found that SMY treatment could modulate the structure and diversity of the gut microbiome in spleen deficiency, and that SMY induced more notable changes, suggesting that it could potentially be the major ingredient of SMY involved in counteracting dysbiosis. However, there is still no systematic and comprehensive interpretation for the association between the effect of SMY in spleen deficiency and its influence on the gut microbiota, warranting further investigation.

## Conclusions

Our findings suggest that SMY may treat spleen deficiency by modulating the gut microbiota, although, further studies are needed to clarify the mechanism by which the regulation of related gut microbiota occurs.

## Data Availability

The datasets used and/or analyzed during the current study are available from the corresponding author on reasonable request.

## Abbreviations

PCR: Polymerase chain reaction
OTUs: Operational taxonomic units
RDP: Ribosomal Database Project
PCoA: Principal coordinates analysis
ANOVA: One-way analysis of variance
ANOSIM: Analysis of similarities
NC: Normal control group
MC: Model control group
SMY: Shengmai Yin group

## References

[1] Wu XN (1998). Current concept of spleen-stomach theory and spleen deficiency syndrome in TCM. World J Gastroenterol.

[2] Peng Y, Zhang S, Liu Z, Ji J, Wu C (2020). Gut microbiota and Chinese medicine syndrome: altered fecal microbiotas in spleen (Pi)-deficient patients. J Tradit Chin Med.

[3] Shi K, Linghang Qu, Li X (2020). Deep-fried atractylodis rhizoma protects against spleen deficiency-induced diarrhea through regulating intestinal inflammatory response and gut microbiota. Int J Mol Sci.

[4] Ma S, Jiang Y, Zhang B (2019). Comparison of the modulatory effect on intestinal microbiota between raw and bran-fried atractylodis rhizoma in the rat model of spleen-deficiency syndrome. Int J Environ Res Public Health.

[5] Chen YL, Fu R, Liu XJ, Liu ZY (2013). Literature research of spleen asthenic syndrome symptoms regularity. China J Chin Med.

[6] Qiu JJ, Liu Z, Zhao P, Wang XJ, Li YC (2017). Gut microbial diversity analysis using Illumina sequencing for functional dyspepsia with liver depression-spleen deficiency syndrome and the interventional Xiaoyaosan in a rat model. World J Gastroenterol.

[7] Wang XM, Li XB, Peng Y (2017). Impact of Qi-invigorating traditional Chinese medicines on intestinal flora: a basis for rational choice of prebiotics. Chin J Nat Med.

[8] Ding HY, Wang BH, Xu Q (2018). The treatment of Classic prescription of shengmaiyin on heart failure: a meta-analysis. Guiding J Tradit Chin Med.

[9] Yuan CJ, Wang HT, Yuan ZY. Ginsenoside Rg1 inhibits myocardial ischaemia and reperfusion injury via HIF-1 α-ERK signaling pathways in a diabetic rat model. 2019. Doi: 10.1691/ph.2019.8858. 10.1691/ph.2019.8858 30961682

[10] Wang J, Feng W, Tang F (2019). Gut microbial transformation, a potential improving factor in the therapeutic activities of four groups of natural compounds isolated from herbal medicines. Fitoterapia.

[11] Juan-Juan L, Zhe Z (2017). Gut microbial diversity analysis using Illumina sequencing for functional dyspepsia with liver depression spleen deficiency syndrome and the interventional Xiaoyaosan in a rat model. World J Gastroenterol.

[12] You Y, Luo L, Chen ZJ (2019). Study on the optimization of the extraction technology of Shengmaiyin Polysaccharide and its regulation effects on intestinal function of spleen deficiency model rats. China Pharmacy.

[13] Ni W, Xin H, Tie L, Mei W (2020). Application of RRLC-QTOF-MS based metabonomics and UPE for investigating Spleen-Qi deficiency syndrome with Panax ginseng treatment. Ethnopharmacology.

[14] Zhou SS, Xu J, Zhu H, Wu J, Xu JD, Yan R (2017). Gut microbiota-involved mechanisms in enhancing systemic exposure of ginsenosides by coexisting polysaccharides in ginseng decoction. Sci Rep.

[15] Shi LL, Li Y, Wang Y, Feng Y (2015). MDG-1, an Ophiopogon polysaccharide, regulate gut microbiota in high-fat diet-induced obese C57BL/6 mice. Int J Biol Macromol.

[16] Zhou WD, Xiang L, Chen ZW, Lu HQ, Luo R, Sun XM (2016). Schisandrae Chinensis fructus polyscaaharide protects against chemotherapy-induced enteritis in mice models. Chin J Exp Tradit Med Formulae.

[17] Shen LB, Qian HN (2005). Summary of experimental research methods of spleen deficiency model. Chin J Inf Tradit Chin Med.

[18] Pang X, Liu YQ, Liu XD, Guan MY, Cai Q (2016). Study on the pharmacodynamic comparison of active part in crude Atractylodes lancea and Atractylodes lancea fried with bran. J China Pharm.

[19] Xue DH, Liu YQ, Cai Q, Liang K, Zheng BY, Li FX, Pang X (2018). Comparison of bran-processed and crude atractylodes lancea effects on spleen deficiency syndrome in rats. Pharmacogn Mag..

[20] Liu F, Liu YJ, Tian CM (2015). Comparative study of effect of rhizoma atractylodis before and after fried with bran on gastrointestinal immune function and hormone of experimental spleen deficiency rats model. Lishizhen Med Mater Med Res.

[21] Zhang L, Liu XY, Xu W, Yang XW (2019). Pharmacokinetics comparison of 15 ginsenosides and 3 aglycones in Ginseng Radix et Rhizoma and Baoyuan decoction using ultra-fast liquid chromatography coupled with triple quadrupole tandem mass spectrometry. Phytomedicine.

[22] Shi LL, Wang Y, Feng Y (2015). Effect of MDG-1, a polysaccharide from Ophiopogon japonicas, on diversity of lactobacillus in diet-induced obese mice. Zhongguo Zhong Yao Za Zhi.

[23] Wang HM, Zhang LP, Chen LR, Liu J (2015). Gastrointestinal motility and spleen deficiency in gastroesophageal reflux disease. Chin J Integr Tradit West Med Digest.

[24] Digestive physiology research group, Department of biology, Beijing Normal University. Establishment of animal model of spleen deficiency syndrome and its essence. J Beij Normal University 1979 (1): 113.

[25] Huang B, Mao Y, Fan L (1983). Animal model of spleen deficiency caused by improper diet and observation of Chinese medicine treatment. Chin J Integr Tradit Western Med.

[26] Chen X, Zhou Y, Fan Y (2001). Preliminary study on standardization of animal model of spleen qi deficiency syndrome. Chin J Tradit Chin Med Pharm.

[27] Zhang L, Mei J, Huang Z (1999). Experimental study on pathogenic mechanism of external dampness. J Tradit Chin Med.

[28] Administration STS (1997). Terminology of clinical diagnosis and treatment of traditional Chinese Medicine.

[29] Wang J, Feng W, Zhang S, Chen L (2019). Ameliorative effect of Atractylodes macrocephala essential oil combined with Panax ginseng total saponins on 5-fluorouracil induced diarrhea is associated with gut microbial modulation. J Ethnopharmacol.

[30] Wang Z (2008). Application of gut-microbiota molecular biomarker in researching active ingredients of Chinese medicine for spleen deficiency treatment.

[31] Li QM, Zhang YJ, Zhang DF, Liu J, Li LJ (2010). Jianpi Zhi xie granule’s effect of regulating micro-ecology of spleen-deficiency and antibiotics-induced dysbiosis mice. Chin J Basic Med Tradit Chin Med.

[32] Lloyd-Price J, Abu-Ali G, Huttenhower C (2016). The healthy human microbiome. Genome Med.

[33] Rios-Covian D, Salazar N, Gueimonde M, Reyes-Gavilan CG (2017). Shaping the metabolism of intestinal *Bacteroides* population through diet to improve human health. Front Microbiol.

[34] Natividad JMM, Verdu EF (2013). Modulation of intestinal barrier by intestinal microbiota: pathological and therapeutic implications. Pharmacol Res.

[35] den Besten G, van Eunen K, Groen AK, den Besten G, van Eunen K, Groen AK (2013). The role of short-chain fatty acids in the interplay between diet, gut microbiota, and host energy metabolism. J Lipid Res.

[36] Bäumler AJ, Sperandio V (2016). Interactions between the microbiota and pathogenic bacteria in the gut. Nature.

[37] Lin L, Wang FY, Tang XD, Ma XX, Yin XL, Shi XS (2017). Effect of Pixu I recipe on cytochrome c oxidase subunit va expression in gastric tissues of FD rats with spleen deficiency. Chin J Exp Tradit Med Formulae.

[38] Zhong ZS, Zhang HY, Zhang W, He GH, Ye ZH, Wang J, Huang SP (2018). Effect of Si Junzitang on CaM-MLCK pathway in rats with spleen deficiency and gastrointestinal motility disorder. Chin J Exp Tradit Med Formulae.

[39] Wang HM, Zhang LP, Chen LR (2015). Gastrointestinal motility and spleen deficiency in gastroesophageal reflux disease. Chin J Integr Tradit West Med Digest.

[40] Louis P, Flint HJ (2017). Formation of propionate and butyrate by the human colonic microbiota. Environ Microbiol.

[41] Rivière A, Selak M, Lantin D, Leroy F, De Vuyst L (2016). Bifidobacteria and butyrate-producing colon bacteria: importance and strategies for their stimulation in the human gut. Front Microbiol.

[42] Derrien M, Vaughan EE, Plugge CM, de Vos WM (2004). Akkermansia municiphila gen. nov., sp. nov., a human intestinal mucin-degrading bacterium. Int J Syst Evol Microbiol.

[43] Saez-Lara MJ, Gomez-Llorente C, Plaza-Diaz J, Gil A (2015). The role of probiotic lactic acid bacteria and bifidobacteria in the prevention and treatment of inflammatory bowel disease and other related diseases: a systematic review of randomized human clinical trials. Biomed Res Int.

[44] Rokana N, Singh R, Mallappa RH, Batish VK, Grover S (2016). Modulation of intestinal barrier function to ameliorate Salmonella infection in mice by oral administration of fermented milks produced with *Lactobacillus plantarum* MTCC 5690 - a probiotic strain of Indian gut origin. J Med Microbiol.

[45] Saulnier DM, Riehle K, Mistretta TA, Maria-Alejandra Diaz MA, Mandal D, Raza S, Weidler EM (2011). Gastrointestinal microbiome signatures of pediatric patients with irritable bowel syndrome. Gastroenterology.

[46] Ju T, Ji YK, Paul S, Benjamin PW (2019). Defining the role of Parasutterella, a previously uncharacterized member of the core gut microbiota. ISME J.

[47] Liu H, Chen X, Hu X (2019). Alterations in the gut microbiome and metabolism with coronary artery disease severity. Microbiome.

